# The liquid biopsy in lung cancer

**DOI:** 10.18632/genesandcancer.127

**Published:** 2016-11

**Authors:** Junaid Ansari, Jungmi W. Yun, Anvesh R. Kompelli, Youmna E. Moufarrej, Jonathan S. Alexander, Guillermo A. Herrera, Rodney E. Shackelford

**Affiliations:** ^1^ Feist Weiller Cancer Center, LSU Health Shreveport, LA, USA; ^2^ Department of Molecular and Cellular Physiology, LSU Health Sciences Center, Shreveport, LA, USA; ^3^ School of Medicine, LSU Health Shreveport, Shreveport, LA, USA; ^4^ Department of Pathology, LSU Health Shreveport, Shreveport, LA, USA

**Keywords:** circulating tumor cells, circulating cell-free tumor DNA, liquid biopsy, tumor educated platelets, epidermal growth factor receptor

## Abstract

The incidence of lung cancer has significantly increased over the last century, largely due to smoking, and remains the most common cause of cancer deaths worldwide. This is often due to lung cancer first presenting at late stages and a lack of curative therapeutic options at these later stages. Delayed diagnoses, inadequate tumor sampling, and lung cancer misdiagnoses are also not uncommon due to the limitations of the tissue biopsy. Our better understanding of the tumor microenvironment and the systemic actions of tumors, combined with the recent advent of the liquid biopsy, may allow molecular diagnostics to be done on circulating tumor markers, particularly circulating tumor DNA. Multiple liquid biopsy molecular methods are presently being examined to determine their efficacy as surrogates to the tumor tissue biopsy. This review will focus on new liquid biopsy technologies and how they may assist in lung cancer detection, diagnosis, and treatment.

## INTRODUCTION

Lung cancer (LC) is the second most commonly diagnosed cancer and remains the leading cause of cancer deaths worldwide, with an estimated 1.8 million new cases in 2012 [1]. Smoking is the main risk factor, with other risk factors being genetic predisposition, and environmental exposures such as radon, arsenic, polycyclic aromatic hydrocarbons, and asbestos [1, 2]. Currently the 5-year overall survival rate for lung cancer is 17-22%, although survival differs with different stages at initial diagnosis. The low survival rate in LC is partially due to only 20% of LCs diagnosed at early stages, with the remaining diagnosed at later stages, often precluding curative treatment [1-3]. When detection methods are used that allow earlier diagnoses, LC survival increases. For example, compared to the standard chest X-ray, spiral computed tomography (CT) reduces LC mortality by 20% due to earlier detection [4]. The high morbidity and mortality associated with LC and the improved survival with earlier detection methods highlights the need for better early detection methods, especially as many patients who achieve an initial remission have recurrences [1-4].

LC is subdivided into small cell LC (SCLC) and non-small cell LC (NSCLC), constituting approximately 15% and 85% of LCs, respectively [5]. NSCLC is further subdivided into adenocarcinoma (AdenoCA), squamous cell carcinoma (SCC), and several less common subtypes [5]. Presently pulmonary AdenoCAs are usually analyzed for epidermal growth factor receptor (EGFR) activating mutations, and ROS1 and anaplastic lymphoma tyrosine kinase (ALK) gene rearrangements, as these molecular changes have targeted therapies that improve treatment outcomes [5-8]. The most common different LC subtypes, their prognoses, and immunohistochemical and molecular markers are summarized in Table 1.

**Table 1:** A summary of the features of the most common lung cancer subtypes, including the prognosis by 5-year survival at different stages, the LC type immunohistochemistries commonly used in diagnosis, common histologic features, and common molecular alterations

NSCLC	Prognosis 5-yr Survival by Stage	Histology	IHC	Genes Mutated	References
Adenocarcinoma ∼60% of NSCLC	IA - 49% IB - 45% IIA - 30% IIA - 30% IIB - 31% IIIA - 14% IIIB - 5% IV - 1%	Glandular differentiation with prominent nucleoli in lepidic, papillary, solid, and/or acinar growth patterns.	CK7+ TTF-1+ Napsin A+ CK20-	p53 Mt 90% KRAS Mt 32.2% EGFR Mt 15% FGFR4 Mt 7% ALK Fus 3.9% BRAF Mt 7% ROS1 Fus 2% c-MET Amp 4% RET Fus 1% HER2 Mt 0.9%	5,7-13,15,16
Squamous Cell Carcinoma 20-30% of NSCLC		Polygonal cells in island or sheets with intercellular bridges, eosinophilic cytoplasm, prominent nucleoli, and may have keratin pearl formation.	p63+, CK5/6+, p40+, CEA+, EMA+ CK7-, TTF-1-	p53 Mt 90% FGFR1 Amp 20% PIK3CA Mt 12% PTEN Mt 10% KRAS Mt 6% EGFR Mt 5% DDR2 Mt 4% BRAF Mt 2%	5,9,17-26
Small Cell Lung Cancer 15-20% of lung cancers 99% occur in smokers	I - 31% II - 19% III - 8% IV - 2%	Round-oval cells with nuclear molding, crush artifact, “salt and pepper” chromatin, absent nucleoli, and brisk miotic rate.	pan-keratin+, TTF-1+, NSE+, CD117+, CD57+ chromogranin+, synaptophysin+	p53 Mt 90% RB1 Mt 60% KRAS Mt 25% RET Fus 5% EGFR Mt 4% PIK3CA Mt 3% ROS1 Fus 1%	27-34

IHC = immunohistochemistry, Mt = mutation, Fus = fusion, Amp = amplification.

Such markers often guide patient treatment options and confer valuable prognostic information. For example, late-stage AdenoCAs with activating EGFR mutations can be treated with the first or second-line anti-EGFR tyrosine kinase inhibitor (TKI) drugs erlotinib, gefitinib, or afatinib. All which show superior response rates and outcomes compared to standard chemotherapy [5, 6]. Similarly, specific chemotherapeutic drugs block the activities of mutated EML-ALK, BRAF, and ROS1 kinases in AdenoCAs, improving clinical outcomes [9-11]. For EML-ALK positive AdenoCAs treatment with the drug crizotinib significantly increases overall patient survival and progression free survival [8]. Additionally, LC often has epigenetic changes, such as increased p16, RASSF1, APC, and H-cadherin gene promotor methylations, which down-regulates tumor suppressor gene expression, promoting cancer progression [12]. Presently new therapies targeting LC epigenetic changes are being developed and some have already shown utility in LC treatment [13].

In LC diagnosis and staging, imaging and diagnostic biopsies play central roles. Currently, CT is often employed in LC diagnosis and staging, but many lung nodules are still found incidentally by chest X-ray [4]. Positron emission tomography (PET) may be a useful modality in the work up of a suspected LC and often allows the distinction of benign from malignant lesions. However, concerns over false positives due to inflammation or false negatives due to small and less metabolically active nodules, or nodules with a low number of tumors cells, are flaws with PET [14].

In order to create a molecular profile of a tumor, a biopsy or several biopsies may be required. Recurrent biopsies are invasive and may miss portions of the tumor that are developing treatment resistance or have acquired new driver mutations [15]. Additionally, in phase 3 IPASS and INTEREST studies, only 42% and 31% of LC patients had tissue available or obtainable, respectively, limiting needed molecular diagnostic studies [16-18]. Lastly, recent studies reveal that malignant tumors have significant molecular heterogeneity, with cells from one portion of a tumor having different mutations than other areas and sometimes sharing only one-third of the mutations throughout the tumor [19]. Therefore minimally invasive modalities that could guide early detection, follow patients regularly, allow early emerging treatment resistance assessment, and identify new driver mutations would be useful in optimizing LC care [20].

Recent work on blood-based biomarker analyses, including circulating cell-free tumor DNA (cfDNA), circulating tumor cells (CTCs), and tumor-educated platelets (TEPs), indicate that these biomarkers may allow earlier LC detection with more frequent monitoring. Additionally, these techniques are less invasive and may provide a more complete LC molecular characterization [20-22]. These markers may also allow earlier minimal residual disease monitoring, as tumor cfDNA can be detected at a 50^6^ tumor cell burden, while PET requires a 10^9^ tumor cell burden for detection [23].

## THE LIQUID BIOPSY AND TUMOR MICRO-AND SYSTEMIC ENVI-RONMENTS

Stromal-tumor interactions, referred to as the tumor microenvironment, play an important role in regulating tumor progression and metastatic potential [21, 22]. These interactions include paracrine/juxtacrine signals, and cytokine, cfDNA, and CTC release. Stromal-tumor interactions additionally lead to abnormal tumor-altered cellular phenotypes consisting of TEPs, tumor-associated neutrophils (TANs), cancer-associated fibroblasts (CAFs), and tumor endothelial cells (TECs) [21-25]. Additionally tumors extrude microvesicles (ES) containing lipids, proteins, DNA, and microRNAs, which modulate tumor behavior and stromal-host responses [22]. Lastly, tumors also create a systemic environment where soluble circulating tumor-derived factors recruit cells from organs, such as the spleen and bone marrow, to the tumor site. These processes often enhance local tumor growth and increase tumor metastatic potential [24]. Several of these circulating tumor-derived substances are easily sampled by a blood draw and can be subjected to molecular analyses. These include cfDNA, CTCs, TEPs, and ES [20,21,23-25].

### cfDNA

Tumor cfDNA was discovered in 1948 and is a promising LC molecular diagnostic target [26, 27]. cfDNA is partially composed of DNA fragments roughly 150 to 1,000 base-pairs long which form a ladder-like electrophoretic pattern of roughly 180 bp multimers, possibly indicating an apoptotic origin [27-29]. However, other studies have shown that a significant portion of tumor-derived cfDNA is smaller than 145 bp, arguing against an apoptotic origin [30, 31]. cfDNA from cancer patients also contains hTERT and hTr gene sequences, which are not found in apoptosis-derived DNA. Other larger cfDNA fragments may be derived from necrotic tumor tissue; however, cfDNA levels fall following radiation therapy, suggesting that tumor necrosis is not a major cfDNA source [32-36]. Interestingly, some studies indicate that cfDNA may be actively secreted and have signaling functions promoting metastases (termed “genometastasis”) [37-39]. For example, Garcia-Olmo *et al.* [38] found that NIH-3T3 murine cells incubated in the cell-free plasma of individuals with KRAS mutation-positive colorectal carcinoma developed KRAS mutations. When these cells were injected into mice, the mice grew tumors with human KRAS mutations, and the same mutations were detected in the murine plasma. Similarly, Trejo-Becerril *et al.* [39] showed that NIH-3T3 cells incubated in cell-free KRAS mutation-positive human serum developed KRAS mutations. When these cells were injected into rats with the colon cancer-initiating carcinogen 1,2-dimethylhydrazine, the rats developed KRAS-positive tumors. Although still under intense investigation, it appears that tumor-derived cfDNA has multiple origins and can act as a signaling molecule [27-39]. These findings are interesting in light of the observation by Jiang *et al*. [40] who found that tumors preferentially release tumor DNA bearing tumor-associated mutations.

Tumor cfDNA is found in blood, lymph, milk, spinal fluid, urine, and saliva and can be nuclear and/or mitochondrial [41]. It is less stable than cfDNA derived from non-tumor cells and is rapidly degraded by plasma nucleases, conferring a short half-life [41-43]. In one study the half-life of colorectal adenocarcinoma cfDNA (following a colon cancer resection) was 114 minutes [43]. LC cfDNA is generally higher in SCLC than in NSCLC and its plasma concentration often increases at higher tumor stages [44-46]. Additionally, Szpechcinski *et al.* [47] used realtime PCR to quantify cfDNA in 40 healthy volunteers, 101 individuals with chronic respiratory inflammation, and 50 stage I-IIIA NSCLC patients. cfDNA was 2.27+/−1.51 ng/ml, 3.36+/−2.07 ng/ml, and 8.02+/−7.81 ng/ml in each group, respectively. Thus, NSCLC cfDNA appears to be primarily from tumor development and not inflammation. Interestingly, LC cfDNA levels also have prognostic value, as patients with lower LC cfDNA levels are less likely to progress. A recent meta-analysis of 22 studies involving 2,581 patients revealed that higher LC cfDNA levels correlated with shorter progression-free and over-all survivals [45-48]. Further studies are required to analyze tumor cfDNA size, origin, possible functions, and clinical uses [27-30].

### cfDNA Preparation

cfDNA is usually obtained by phlebotomy and should be processed rapidly due to its short half-life [49,50]. Often blood is collected into an EDTA tube (Vacutainer), which prevents clotting and inhibits DNase I activity [51]. Plasma is most often used as clotting releases benign leukocyte DNA, diluting tumor cfDNA [52]. This DNA can be purified by several different methods, with the QIAamp DNA Blood Mini Kit (Qiagen) commonly used [53]. Newer techniques are being developed to improve cfDNA isolation and preservation. For example, Jeon *et al.* [54] developed a free-standing 3D array of gold nanowires coated with a conducting polymer which could recover ≥10 pg/mL lung and breast cancer cfDNA with a high yield and purity.

### cfDNA Analysis Techniques

Tumor cfDNA is prepared and subsequently analyzed by a variety of techniques, some of which can interrogate the entire exome or genome, while others target specific genes. Each technique has specific advantages and disadvantages, and includes digital PCR (dPCR), scorpion amplification refractory mutation system (ARMS), peptide nucleic acid-locked clamp PCR (PNA-LNA), next generation DNA sequencing methods (NGS), and epigenetic analyses. Tumor cfDNA analysis can be challenging as it can make up as little as 0.01% of the total cfDNA pool. Tumor cfDNA can be present at low ng/ml concentrations, is less stable than non-tumor cfDNA, and is often degraded [27-36,41-43,47,48,55]. Additionally only 0.004% of the LC genome is regularly mutated, making the relevant fraction of cfDNA to be analyzed quite small [55]. Several of the more common cfDNA analysis techniques and their advantages and disadvantages are summarized in Table 2.

**Table 2:** A summary of the more commonly used techniques to analyze cfDNA. The different techniques are described, and the specific advantages and disadvantages, and relative costs of each technique are briefly summarized

Analysis Technique	Description	Advantages	Disadvantages	Refs
**Conventional Real-Time qPCR**	DNA is amplified by repeated cycles of DNA, primer, and probe thermal denaturation, annealing, and DNA polymerization with a heat-tolerant DNA polymerase.	Low cost, relatively easily implemented.	Low sensitivity for cfDNA, only interrogates DNA for areas between the primer sequences.	[56-58]
**Scorpion ARMS PCR**	A bi-functional PCR primer covalently linked to a probe with closely associated fluorophore and a quencher. Amplification will only happen if the 3′ primer nucleotides match the target sequence. During the PCR reaction, the fluorophore and quencher become separate, giving a detectable fluorescence.	Relatively low cost and high sensitivity.	Only identifies the specific sequences the probes are designed to detect.	[59-61]
**PNA-LNA PCR**	Composed of an uncharged polyamide backbone with attached bases that hybridizes to ssDNA with high affinity allowing probes enhanced binding to dilute cfDNA sequences than standard PCR DNA probes.	High sensitivity, lower cost.	Only identifies the specific sequences the probes are designed to detect.	[62,63]
**NGS**	DNA is fragmented into millions of short segments, ligated to DNA adaptor molecules, segregated on a solid matrix, and sequenced in parallel by labeled nucleotides addition, and bioinformatically aligned into a genomic sequence.	Can target specific sequences, the exome, entire genome, detect all sequence variations, rearrangements, copy number changes, and often gene fusions.	High cost, complex to implement, analysis complex and requires bioinformatics analysis.	[19,64-66]
**dPCR**	Partitions cfDNA into thousands of parallel individual PCR reactions. Signal detection means the target sequence is present.	High sensitivity, can analyze multiple analytes simultaneously.	Only identifies the specific sequences the probes are designed to detect.	[67,68]

## APPLICATIONS OF CFDNA LC ANALYSIS AND TREATMENT

cfDNA analysis has multiple applications in LC treatment, including analyzing tumor molecular heterogeneity, monitoring disease burden and prognosis, and the early detection of emerging therapy resistance. Here we will briefly discuss these analyses.

### cfDNA and Tumor Molecular Heterogeneity

Alegre *et al.* [69] used dPCR to analyze EGFR mutation positive cfDNA in LC patients being treated with EGFR inhibitors. Interestingly, dPCR cfDNA EGFR analysis revealed EGFR mutations not detected by tumor biopsy analysis in 34% of patients examined. Although a single study with a low (*N* = 36) patient number, it indicates that the liquid biopsy can reveal information about tumor molecular heterogeneity not found by tissue biopsy.

### cfDNA and lung cancer disease burden monitoring and prognosis

KRAS mutation positive pulmonary AdenoCAs are presently not treatable, although targeted therapies are being developed [70]. KRAS mutation positive tumors often have a poor prognosis and are associated with a high mutation burden [72]. Guibert *et al.* [71] used dPRC to serially analyze KRAS positive AdenoCA cfDNA in 34 patients with tumors carrying KRAS mutations identified by tumor tissue PCR analyses. Patients who progressed had an initial average cfDNA level of 947 KRAS mutated alleles/ml, while those whose disease was controlled averaged 147/ml. Additionally, higher mutated KRAS cfDNA allele levels correlated with poor therapy responses (71).

Sozzi *et al.* [73] analyzed 43 healthy individuals and 84 NSCLC patients and found that plasma cfDNA was higher in NSCLC patients (including stage I patients) with an average of 318 ng/ml compared to 18 ng/ml in healthy controls. Where follow-up was performed, NSCLC patients' cfDNA averaged 345 ng/ml before tumor resection and fell to 34 ng/ml post-surgery. cfDNA showed a trend towards reduction in relapse-free patients, but showed a 2-20-fold increases in relapsed patients. In a later study, 1,035 heavy smokers were followed for 5-years with spiral CT combined with plasma DNA PCR analysis [46]. Of 38 individuals who developed LC, higher plasma cfDNA was associated with a poorer 5-year survival.

Newman *et al.* [55] used the CAPP-Seq technique to quantify cfDNA in NSCLC patients and compared cfDNA levels to CT and PET tumor imaging. cfDNA levels significantly correlated with CT and PET measured tumor volume (R_2_ = 0.89, *P* = 0.0002). In the same study, 3 patients' cfDNA was followed during NSCLC therapy and lower or higher cfDNA plasma concentrations correlated with better or worse outcomes, respectively. While other studies have failed to show a correlation between cfDNA concentrations and PET-measured tumor volumes, it appears that careful cfDNA analysis correlates with LC tumor burden and can have prognostic value [46, 55, 71, 72].

Activating EGFR receptor mutations are found in about 15% of lung AdenoCAs, 90% of which are either the L858R point mutation or in-frame deletions of exon 19 [5, 6, 75-77]. These AdenoCAs are often treated with erlotinib, gefitinib, or afatinib [5, 6]. Most patients receiving these drugs will develop drug resistance within one year. Approximately 50% of this resistance is due to EGFR T790M mutation emergence [78]. Some evidence indicates that T790M may be present at low levels in EGFR mutated AdenoCAs prior to EGFR drug therapy [79, 80]. Wang *et al.* [81] used dPCR and ARMS to analyze T790M cfDNA mutations in 135 TKI-treated lung AdenoCAs patients who had progression-free survival on the TKIs for over six months. dPCR and ARMS identified T790M in 31.3% and 5.5% of patients prior to TKI therapy, respectively, demonstrating that dPCR was a more sensitive test. Post TKI therapy, the same tests showed T790M in 43.0% and 25.2% of patients, respectively. Patients with high pre-TKI therapy T790M levels had a poorer progression-free and overall survival, demonstrating prognostic value.

### cfDNA and lung cancer therapy resistance monitoring

Sorensen *et al.* [82] used allele-specific PCR to quantify 42 different erlotinib-sensitive EGFR mutations in the plasma of 23 patients with advanced AdenoCAs. Sampling was performed before erlotinib treatment and then done sequentially with erlotinib treatment. Ninety-six percent (22/23) of patients showed a reduction in the sensitizing mutations (L858R or ex19Del) upon erlotinib treatment initiation. Initially no patients carried the T790M mutation. With treatment, 39% of the patients developed the T790M mutation with a concomitant increase in erlotinib-sensitive EGFR mutations. Interestingly, T790M elevations appeared up to 344 days before clinical LC progression was evident (range 15-344 days). Thus, serial monitoring for T790M may allow the earlier detection of resistance-related LC progression. Detecting T790M is also important as the TKI TAGRISSO is now used clinically to target T790M positive NSCLC [83].

Miyazawa *et al.* [63] used a PNA-LNA probe to examine T790M in 151 gefitinib-treated NSCLC from 17 sputums, 63 bronchial washings, 3 transbronchial biopsies, 4 needle aspiration biopsies, 17 pleural effusions, and 1 pericardial effusion. Initially no patients carried T790M, but 4 patients who became resistant to gefitinib developed T790M. Oxnard *et al.* [84] used dPCR to examine cfDNA in patients with advanced NSCLC carrying exon 19 deletions treated with erlotinib. Nine patients without an initial T790M mutation were treated with erlotinib and 8 had a complete “plasma response”, or a complete loss of mutant EGFR cfDNA. In 6 patients the cfDNA exon 19 deletion EGFR mutation concomitantly rose with T790M 4-12 weeks prior to RECIST progression. The remaining patients had indolent asymptomatic progression in the chest only and remained on erlotinib. Further, Watanabe *et al.* [85] examined 373 NSCLC patient tumor samples with EGFR-activating mutations for T790M via ultra-sensitive dPCR. T790M was found in 79.9% of the patients and the mutation was associated with larger tumor volumes. This data, combined with the previously cited studies, indicates that LC often carries T790M and cfDNA analysis may have value in monitoring disease burden, disease progression, and drug resistance emergence [83].

### CTCs

First identified by autopsy in 1869, CTCs arise from solid tumors and circulate in the blood and/or lymphatic vessels [86]. CTCs are heterogeneous cell populations that circulate as single cells or as tumor cell clusters, and are assumed to contain subpopulations with metastatic potential or the ability to re-circulate back to the tumor (termed “tumor self-seeding/cross seeding”) [86-90]. High baseline CTCs concentrations at initial diagnosis and/or high CTCs following one cycle of chemotherapy confer a poor LC prognosis [91-94]. LC CTCs may increase at higher tumor stages and are often higher in SCLC than in NSCLC [92, 95]. Measurements using murine models indicate that for each gram of tumor, about 10^6^ CTCs are released per day [96]. Despite this, CTC blood concentrations are often low at 1-10 CTCs per ml of blood, which contains 10^6^-10^7^ white cells [91-96]. Additionally, several studies of LC patients' CTCs revealed that in 15-70% of LC patients CTCs are not detectable [55,56,58].

Due to their low concentrations, CTC analysis often requires an initial enrichment step. While there are many different enrichment techniques, they often employ isolation either based on cell-specific surface markers (CD45-, EpCAM+, and cytokeratins 8, 18, and/or 19) or features often unique to CTCs, such as a larger size (10μm for leukocytes vs. 15μm for many CTCs) [97-100]. Detecting EpCAM+ CTCs in NSCLC is likely to miss many CTCs, as NSCLC CTCs undergo an epithelial-mesenchymal transformation becoming EpCAM- and vimentin+ [91, 101, 102]. Farace *et al.* [101] examined the abilities of the CellSearch and Isolation by SizE of Tumor cells (ISET) CTC isolation systems to detect NSCLC CTCs in the blood of 20 patients. The CellSearch isolates EpCAM+, CD45-CTCs, while Isolation by ISET isolates CTCs based on cell size. The two methods had a low 20% concordance, indicating isolating EpCAM+ NSCLC CTCs may miss many CTCs. Other studies revealed that size filtration followed by cytologic characterization gave a more efficient NSCLC CTC isolation, allowing the detection of EML-ALK and ROS1 rearrangements in CTCs [94,103,104]. Other CTC isolation techniques include CTC microchips, automated microscopy systems, enzyme-linked epithelial immunospot assays, and reverse-transcriptase PCR [101, 105-108]. Recently CTC isolation techniques have been developed that can isolate CTCs at very low concentrations. For example, Yoon *et al.* [109] used functionalized graphene oxide nanosheets on a patterned gold surface to capture CTCs at 3-5 cells/ml with 73% efficiency. Due to the often low CTC cell yield, cell numbers have been increased by *in vitro* culture or engrafted into immunocompromised mice, increasing the sample volume for analysis [110, 111].

Once isolated LC CTCs can be analyzed like other pulmonary tissue. Maheswaran *et al.* [112] used a microfluidic device containing microposts coated with antibodies against epithelial cells followed by allele-specific polymerase-chain-reaction EGFR mutation amplification in NSCLC patients. L858R, del 19, and T790M mutations were detected in the CTCs. Interestingly patients who were pretreatment-positive for T790M CTCs had a progression-free survival of 7.7 months vs. 16.5 for those who were initially T790M negative. Additionally, serial analysis of CTCs revealed that CTC levels correlate with the radiographic tumor response and increasing CTCs were associated with tumor progression and in some cases the emergence of new EGFR mutations. Aieta *et al.* [113] followed EML-ALK levels in a NSCLC patient's CTCs and found a CTC reduction upon successful crizotinib treatment. Similarly, Ilie *et al.* [114] examined 87 lung AdenoCA patients by ALK FISH analysis and found 5 cases positive by both tissue biopsy and CTC analysis. Lastly, Guibert *et al.* [115] detected KRAS mutations by dPCR in 34% of CTCs in 32 NSCLC patients. In the same study, cfDNA analysis allowed detection in 82% of the same patients, demonstrating the superior sensitivity of cfDNA analysis. These studies demonstrate that CTC analysis can guide LC therapy, monitor treatment resistance emergence, and provide prognostic information. Currently there have been nearly 400 clinical trials examining LC CTCs, but so far, LC CTCs are rarely used in clinical practice (see “ClinicalTrials.gov”).

### TEPs

At all times approximately 75-200 × 10^10^ platelets circulate in the bloodstream of healthy individuals, making them the second most abundant peripheral formed blood element [116]. Platelets play a central role in thrombosis and wound healing, and regulate systemic and local responses to malignancy [117]. Tumor cells can transfer tumor-associated biomolecules to platelets, an event termed “tumor education” As a result platelet RNA and protein profiles are altered to promote tumor growth and increase tumor metastatic potential [117, 118]. Best *et al.* [117] used RNA sequencing to analyze 283 patient platelet samples from 55 healthy individuals and 228 patients with NSCLC, glioblastoma, colon, pancreatic, hepatobiliary, and breast cancers. The analysis was 96% accurate in distinguishing healthy individuals from those with cancer, 71% accurate in identifying the specific cancer subtypes, and correctly identified EGFR, KRAS, and PIK3CA mutations.

Nilsson *et al.* [118] analyzed 77 NSCLC patients, 32 of whom carried EML-ALK fusions. RT-PCR of platelet RNA had 65% sensitivity and 100% specificity for EML-ALK detection. Interestingly, analysis of circulating RNAs in the same patients had a 21% sensitivity and 100% specificity, likely reflecting lower RNA stability in the latter samples. In a 29 patient subset treated with crizotinib, progression-free survival was 3.7 months in individuals with EML-ALK+ platelets and 16 months for those with EML-ALK-platelets. Several crizotinib-treated patients initially EML-ALK+ became negative following treatment and one patient serially followed over 30 months showed crizotinib-resistance measured by increased platelet EML-ALK 2 months before progression was detected by PET-CT. Although preliminary, these studies indicate that TEPs may be useful in LC diagnostics, particularly as they are more abundant than CTCs [119].

### TECs

TECs line tumor blood vessels and are morphologically and biochemically distinct from normal endothelial cells (ECs). They often show unusual tortuous shapes and a chaotic organization not seen in benign tissues. TECs can have projections that extend into the vessel lumen and impair normal barrier functions, allowing the leakage and pooling of erythrocytes [121]. TECs show radically different gene expression patterns than ECs in non-cancerous tissues. For example, Maishi *et al.* [25] reported a role for TECs in tumor metastasis, demonstrating a bidirectional crosstalk between tumor cells and TECs that regulated tumor metastasis. Using a murine cancer model, TECs isolated from highly metastatic tumors, accelerated metastasis when implanted in animals with low metastatic potential tumors. Molecular analysis of the TECs promoting metastasis revealed decreased biglycan promoter methylation with increased biglycan expression. Biglycan activates both NF-κB and extracellular signal-regulated kinase1/2 in the tumor cells, promoting metastasis. Examination of the PrognoScan database, a large collection of publicly available cancer microarray datasets with clinical correlation, revealed increased biglycan levels correlate with increased metastasis in human tumors, including LC [25]. TECs will require extensive research before having clinical applications, however present data supports a role for them in tumor metastasis.

### ES

ES are released by exocytosis and consist of membranous particles occurring in many body fluids, including blood, ascites, and pleural effusions [123-125]. Two classes of ES have been identified; exosomes (30-120 nm) and microvesicles (100-10^3^ nm). ES are enriched for several proteins, and contain mRNA, miRNA, tRNA, ssDNA, mitochondrial DNA, and dsDNA [125, 126]. Tumor-released ES appear to facilitate tumor growth by activating benign cells towards tumor growth-promoting phenotypes [127]. NSCLC secretes ES and Lin *et al.* [128] demonstrated that the miRNA patterns are different between the pleural effusions in pulmonary tuberculosis, pneumoniae, and NSCLC, making them clinically distinguishable [128-130]. Sandfeld-Paulsen *et al.* [130] examined ES in 276 NSCLC patients captured with antibodies to general ES marker proteins. Forty-nine proteins attached to the exosomal membranes were evaluated based on their presence and concentration, and correlated with over-all patient survival. Of the 49 proteins, increased NY-ESO-1 was present in 50% of the patients and was a highly significant inferior survival prognostic biomarker. Brinkmann *et al.* [131] purified ES RNA from NSCLC patients and subjected it to RT-qPCR for EML4-ALK transcripts. EML4-ALK transcripts were detected in 70% of cases known to carry the fusion by previous tissue biopsy analysis. Similarly Krug *et al.* [132] used NGS to analyze activating EGFR mutations and the T790M EGFR mutations in blood ES from 54 NSCLC patients who had these mutations previously identified by tumor tissue analysis. A 96% concordance between the tumor biopsy results and ES and cfDNA NGS results was found for the activating EGFR mutations and 86% for the T790M mutation. Taken together these results indicate that ES analysis may have value in LC diagnostics.

## CONCLUSIONS AND FUTURE DIRECTIONS

Liquid biopsy approaches hold great potential in LC cancer diagnostics. cfDNA, CTCs, TEPs, TECs, and tumor ES all show promise, although few are currently used clinically [25-48, 69-81,112-115,117-119,128-132]. Presently several challenges must be met before the liquid biopsy can enter clinical practice, including 1) optimizing and standardizing sample gathering, 2) implementing uniform analytical procedures, 3) identifying the circulating analyte(s) most likely to yield useful information, and 4) performing the sufficiently large multi-center clinical trials necessary for validating specific analysis protocols [133]. Additionally, as many circulating analytes occur at low concentrations or are “rare events”, maximizing test sensitivity will be a challenge. Protocols such as those employed by Newman *et al.* [55], which increases assay sensitivity by targeting the specific mutations within LC, will likely prove useful. With time, the technical challenges of the liquid biopsy will likely be solved and it will become an important tool in LC diagnostics and treatment.
